# Neuroprotective Profile of Edible Flowers of Borage (*Borago officinalis* L.) in Two Different Models: *Caenorhabditis elegans* and Neuro-2a Cells

**DOI:** 10.3390/antiox11071244

**Published:** 2022-06-24

**Authors:** Cristina Moliner, Guillermo Cásedas, Lillian Barros, Tiane C. Finimundy, Carlota Gómez-Rincón, Víctor López

**Affiliations:** 1 Department of Pharmacy, Faculty of Health Sciences, Universidad San Jorge, 50830 Zaragoza, Spain; acmoliner@usj.es (C.M.); gcasedas@usj.es (G.C.); cgomez@usj.es (C.G.-R.); 2 Instituto Agroalimentario de Aragón-IA2, CITA-Universidad de Zaragoza, 50059 Zaragoza, Spain; 3 Centro de Investigação de Montanha (CIMO), Instituto Politécnico de Bragança, Campus de Santa Apolónia, 5300-253 Bragança, Portugal; lillian@ipb.pt (L.B.); tiane@ipb.pt (T.C.F.)

**Keywords:** *Borago officinalis*, PUFA, antioxidant, neuroprotection, *C. elegans*, Neuro-2a, functional foods

## Abstract

The flowers of *Borago officinalis* L. (Boraginaceae), commonly known as borage, are widely used as a culinary ingredient. The aim of this study was to assess the potential benefits of fresh borage flower extract related to antioxidant, neuroprotective and anti-aging properties. The extract was obtained by Soxhlet extraction with ethanol as a solvent, and fatty acids were detected by GC-FID. The antioxidant activity was evaluated in vitro through the DPPH, FRAP and ORAC assays. Regarding the fatty acid (FA) composition, the extract showed high amounts of polyunsaturated FA. The Neuro-2a cell line was used to determine the cytoprotective capacity of the extract subjected to oxidative stress (H_2_O_2_). Moreover, the model organism *Caenorhabditis elegans* was used to assess antioxidant activity, delayed ageing as well as cytoprotection and reduced β-amyloid toxicity. Cells treated with the extract and H_2_O_2_ showed a better response to oxidative stress than the control group, particularly in terms of mitochondrial activity (MTT assay), redox state (ROS formation) and the activity of antioxidant enzymes (catalase and superoxide dismutase). *B. officinalis* flower extract showed promising antioxidant activity in the selected models, without causing toxicity. Hence, the results obtained support the antioxidant properties of borage flowers in different bioassays using living organisms.

## 1. Introduction

*Borago officinalis* L. (Boraginaceae), commonly known as borage, is an annually flowering, herbaceous plant native to the Mediterranean region (Italy, Spain, Middle East, etc.). This species is widely cultivated for consumption of its leaves and flowers, but also for its therapeutic, ornamental and cosmetic uses [1]. Borage flowers have gained interest due to the renewed popularity of edible flowers among consumers. Beyond their aesthetic value, edible flowers are an interesting source of bioactive compounds with positive health-promoting properties. However, this species may contain small amounts of pyrrolizidine alkaloids [2,3,4]. In Europe, there is a long-established use of borage as a mood enhancer, and most of the recent studies of this plant are focused on the seeds, due to the high content of gamma linolenic acids, which have shown anti-inflammatory and anti-fibrotic effects [5,6].

It is well known that neurodegenerative diseases and inflammatory and cardiovascular disorders have a common mechanism, the production of reactive oxygen species (ROS). Phenolic extracts of plant materials have been shown to neutralize free radicals in various model systems and antioxidant activity [7,8], but few researchers have identified the antioxidant profile of borage [9,10]. To improve the knowledge of this edible plant and confirm the absence of toxicity, other techniques have been used. As the literature shows, neuronal and *C. elegans* models subjected to oxidative stress are suitable for this purpose.

Borage extracts also modulate signaling pathways related to skin photoaging by downregulating collagen degradation and stimulating collagen synthesis, its oil being a beneficial product for eczema and dermatological applications [11].

The aim of this study is to assess the antioxidant and neuroprotective activity in vitro and in vivo, using different methodologies and bioassays in order to establish a scientific basis for future human studies.

## 2. Materials and Methods

### 2.1. Standards and Reagents

AAPH (2,2′-azobis(2-methyl-propionamidine)-di- hydrochloride), BSA (bovine serum albumin), DCFH-DA(2′,7′-dichloro-dihy-drofluorescein diacetate), DPPH (2,2-diphenyl-1-picrylhydrazyl), hydrogen peroxide, MTT (3-(4,5-Dimethyl-2-thiazolyl)-2,5-diphenyltetrazolium bromide), TBA (thiobarbituric acid), TCA (trichloroacetic acid), TPTZ (2,4,6-tris(2-pyridyl)-s-triazine), tris-HCl, Trolox (6-hydroxy-2,5,7,8-tetra-methylchromane-2-carboxylic acid) and pyrogallol were purchased from Sigma-Aldrich (St. Louis, MO, USA). DMEM (Dulbecco’s modified Eagle’s medium), FBS (fetal bovine serum) and PBS (phosphate-buffered saline) were acquired from Gibco (Invitrogen, Paisley, UK). DNTB (5,5′-dithiobis(2-nitrobenzoic acid)) and juglone (5-hydroxy-1,4-naphthoquinone) were from Alfa Aesar (Ward Hill, MA, USA), while Folin–Cicolteau reagent was purchased from Chem-Lab (Zeldelgem, Belgium). All other general laboratory reagents were purchased from Panreac Química S.L.U. (Barcelona, Spain). Water was treated in a Milli-Q water purification system (TGI Pure Water Systems, Greenville, SC, USA).

### 2.2. Plant Material and Preparation of Extract

Fresh flowers of *Borago officinalis* were provided by Innoflower S.L. (Zaragoza, Spain). The extract was obtained with ethanol as a solvent, using a Soxhlet apparatus at an extraction temperature between 80 °C and 85 °C for 4 h. The solvent was removed from the extract with a rotary flash evaporator and the resulting extract was preserved at −20 °C until its use.

### 2.3. Fatty Acid Composition

Fatty acids were determined after a trans-esterification procedure, as described previously by Barros et al. [12], using GC-FID (DANI model GC 1000 instrument, Contone, Switzerland) with a Macherey–Nagel (Düren, Germany) column (50% cyanopropyl-methyl–50% phenylmethyl polysiloxane, 30 m 0.32 mm i.d. 0.25 m df). The program used was as follows: the initial temperature of the column was 50 °C, held for 2 min, followed by a 30 °C/min ramp to 125 °C, 5 °C/min ramp to 160 °C, 20 °C/min ramp to 180 °C, 3 °C/min ramp to 200 °C, 20 °C/min ramp to 220 °C and held for 15 min. The carrier gas (hydrogen) flow rate was 4.0 mL/min, measured at 50 °C. Split injection (1:40) was carried out at 250 °C. Fatty acid identification was performed by comparing the relative retention times of FAME (fatty acid methyl esters, reference standard mixture 37, Sigma-Aldrich, St. Louis, MO, USA) peaks from samples with standards. The results were recorded and processed using CSW 1.7 software (DataApex 1.7). The relative percentage of each fatty acid identification was determined by comparing the relative retention times from samples with standards. The calculated oxidizability value (COX) of the borago flower extract was calculated by applying the formula proposed by Fatemi and Hammond [13] (Equation (1)):

(1) $$\mathrm{COX}=\frac{C18:1+10.3xC18:2+21.6xC18:3}{100}$$

The atherogenic index (*AI*) of the borago flower extract was evaluated based on Ulbricht and Southgate [14] (Equation (2)):

(2) $$AI=\frac{C12:0+4xC14:0+C16:0}{\Sigma MUFA+\Sigma \left(\omega 3\right)+\Sigma \left(\omega 6\right)}$$

### 2.4. In Vitro Antioxidant Activity Assays

#### 2.4.1. DPPH Scavenging Activity

Determination of radical scavenging activity (RSA) was achieved following the protocol described by Lopez et al., with minor modifications [15]. Briefly, 150 µL of a DPPH ethanolic solution (0.04 mg/mL) was added to 150 µL of different extract dilutions in ethanol. After 30 min of incubation in darkness, the absorbance was measured at 517 nm. Radical scavenging activity was calculated as (Equation (3)):

%RSA = ((Abs_control_ − Abs_sample_)/Abs_control_) × 100 (3)

where the Abs_control_ is the absorbance of DPPH^·^ without extract (control) and the Abs_sample_ is the absorbance of DPPH· with the extracts.

#### 2.4.2. Ferric Reducing Antioxidant Power (FRAP) Assay

Ferric reducing ability was assessed according to the method of Pulido et al. [16]. The FRAP reagent was freshly prepared by mixing TPTZ (10 mmol/L in 40 mmol/L HCl), FeCl_3_·6H_2_O (20 mmol/L), and sodium acetate buffer (300 mmol/L, pH 3.6) in a volume ratio of 1:1:10, respectively. Then, 30 μL of flower extract (1 mg/mL) was mixed with 90 μL of distilled water and 900 μL of FRAP reagent. The absorbance of the reaction mixture was measured at 595 nm after incubation for 30 min at 37 °C. The result of the assay was calculated from a standard curve of FeSO_4_·7H_2_O and expressed as μmol Fe^2+^/g extract.

#### 2.4.3. Oxygen Radical Antioxidant Capacity (ORAC) Assay

To evaluate the capacity of the extract to scavenge peroxyl radicals, the ORAC assay was conducted as described by Davalos [17]. Each well contained 20 μL of a dilution of extract or trolox standard mixed with 120 μL of fluorescein (70 mM). Then, 60 μL of AAHH (12 nM) was added and fluorescence was recorded every 70 s for 93 min at 485 nm and 520 nm (excitation/emission, respectively). The area under the curve (AUC) was calculated. Standard curve was constructed using trolox; therefore, results were expressed as μmol trolox equivalents (TE)/mg extract.

### 2.5. Neuro-2a Cell Assays

#### 2.5.1. Cell Culture and Treatments

Mouse neuroblastoma cells (N2a cells; ATCC^®^ CCL-131 ^TM^) were obtained from the American Type Culture Collection (ATCC, Manassas, VA, USA). The cells were cultured in DMEM supplemented with 10% FBS and 1% penicillin–streptomycin and maintained in an incubator at 37 °C, 5% CO_2_, and in a humidified atmosphere.

The treatment was carried out using borago flower extract diluted in sterilized PBS. The sample was filtered with a 0.22 μm syringe filter. Oxidative stress was induced by exposing cells to 300 μM H_2_O_2_ for 1 h.

#### 2.5.2. Cell Viability Assay

The cytotoxicity of the extract was evaluated by using the MTT-based colorimetric method [18]. Briefly, the cells were seeded in 96-well plates at 1 × 10^4^ cells/well. After 24 h, cells were treated with various concentrations of the extract for 24 h. On the next day, oxidative stress was induced with 300 μM H_2_O_2_ for 60 min. Then, 100 μL of MTT stock solution (2 mg/mL in DMEM) was added to each well and the plate was incubated for 3 h. Finally, MTT solution was removed and replaced with 100 μL of DMSO. The absorbance was read at 550 nm in a Synergy H1 Hybrid Multi-Mode Reader (Biotek, Bad Friedrichshall, Germany). This assay was performed over four days and different weeks and passages.

The cell viability was calculated according to the following formula (Equation (4)):

Cell viability (%) = Abs sample/Abs control × 100(4)

#### 2.5.3. Intracellular ROS Production Assay

The production of intracellular reactive oxygen species was determined using DCFH-DA. Neuro-2a cells were seeded in a 96-well plate for 24 h. After this, the medium was removed and PBS (0,18% glucose) and DCFH-DA (2,7-dichlorodihydrofluorescein diacetate, 0.01 M) were added for 30 min at 37 °C [19]. Cells were washed twice with new PBS and supplemented with *Borago officinalis* L. extract (15–250 µg/mL) and H_2_O_2_ (300 µM). The absorbance was checked at 480 nm (excitation) and 520 nm (emission) wavelengths. The kinetic was performed over 90 min in a Synergy H1 Hybrid Multi-Mode Reader (Winooski, VT, USA). Results were represented as a percentage of intracellular ROS production (100% of control).

#### 2.5.4. Endogenous Antioxidant Enzymes

Protein content of treated Neuro-2a cells was determined by the bicinchoninic acid (BCA) assay in order to normalize each pellet by using a lysis buffer with proteinase inhibitors for 20 min (ethylenediaminetetraacetic acid (EDTA) 1 mM, Tris 25 mM, NaCl 150 mM; leupeptin, pepstatin, phenylmethylsulfonyl fluoride, and 0.1% Triton; pH = 7.4).

Finally, supernatants were stored for antioxidant enzyme determination.

Catalase absorbance was calculated following a 30 sec kinetic at 240 nm; 1970 µL of hydrogen peroxide (15 mM; sodium phosphate buffer pH = 7.5) and 30 µL supernatant were mixed in a in a quartz cuvette, using a Shimadzu Spectrophotometer UV-1800 (Duisburg, Germany) [20]. The activity of the enzyme was calculated as (Equation (5)):

Catalase activity = ((DAbs/min) × 2 × F)/(0.0436 ×Vs × C)(5)

43.6 mL nmol^−1^ cm^−1^: molar extinction coefficient of H_2_O_2_;

F: dilution factor;

C: protein concentration (mg/mL);

Vs: sample volume (mL);

DAbs/min: activity of the kinetic.

The absorbance of superoxide dismutase (SOD) was determined at 420 nm for one minute by mixing 1555 µL of Tris–DTPA buffer (50 mM, pH 8.2), 20 µL of pyrogallol (23.78 mM) diluted in HCl (10 mM), and 25 µL total cell extract in a quartz cuvette. This order is required due to the rapid oxidation of pyrogallol [20]. A Shimadzu Spectrophotometer UV-1800 (Duisburg, Germany) was required for the assay. The activity of SOD was expressed as (Equation (6)):

% Inhibition = (DAbs control − DAbs sample)/(DAbs control) × 100 SOD activity = (% Inhibition × 2 × F)/(50 × Vs × C)(6)

### 2.6. C. elegans Assays

#### 2.6.1. Nematode Strain and Maintenance

The following nematode strains were used in this study: N2 (wild type), SS104 glp-4 (bn2), and CL4176 smg-1(cc546). Worms were maintained on Nematode Growth Medium (NGM) agar plates seeded with a lawn of *E. coli* OP50 (Caenorrhabditis Genetics Center, Minneapolis, MN, USA), according to standardized protocols [21]. N2 was propagated at 20 °C, while transgenic CL4176 and SS104 were maintained at 16 °C. The worm and bacterial strains used were provided by Caenorhabditis Genetics Center (CGC, Minneapolis, MN, USA).

#### 2.6.2. Oxidative Stress Resistance Assay

Oxidative stress in nematodes was induced with juglone following the procedure described by Surco-Laos, with modifications [22]. L1 synchronized using alkaline bleach solution were transferred to NGM agar plates containing different concentrations of extract or in the absence of it and cultivated at 20 °C. Once they reached L4, worms were transferred to NGM agar containing 150 μM juglone. After 24 h of exposure at 20 °C, the numbers of dead and alive nematodes were recorded. Worms were scored as dead if they did not respond to touch stimulus with a platinum wire. The results were expressed as a percentage of survival rate (Equation (7)):

% Survival Rate = Number of alive worms/Total number of worms × 100 Assays were performed with at least 120 nematodes per treatment.(7)

#### 2.6.3. Lifespan Assay

Lifespan assay was performed using *C. elegans* SS104, a temperature-sensitive sterile strain, as described by Virk et al. [23]. L1 obtained by egg laying were grown at 16 °C for 72 h. At the beginning of the L4 stage, the temperature was upshifted to 25 °C to induce sterility in worms. A total of 25 worms were transferred to NGM agar plates containing different concentrations of extract or in the absence of it on the first day of adulthood, which was counted as day 0. At least 100 worms were used per condition. Nematodes were transferred to fresh plates every 7 days and survival was scored every 2–3 days from the seventh day. The scoring method was the same as that used for the oxidative stress assay (Section 2.6.2.)

#### 2.6.4. Paralysis Assay

The neuroprotective effect of the extract was evaluated as protection against beta amyloid toxicity in *C. elegans* CL4176 [24]. CL4176 is a transgenic strain with the temperature-inducible muscle expression of human beta amyloid peptide. The accumulation of beta amyloid peptide causes the paralysis of the worms.

L1 synchronized by egg laying were placed on plates containing different concentrations of extract, or in the absence of it, at 16 °C. After 48 h of incubation, the temperature was upshifted to 25 °C to induce transgene expression. Then, 44 h later, the body movement of the worms was scored for 72 h. Nematodes were considered paralyzed if there was no response or they only moved their head after prodding with a platinum wire. For each assay, at least 100 nematodes were studied per treatment.

### 2.7. Statistics

All experiments were performed at least three times. All data were analyzed using GraphPad Prism v.6 and results were expressed as mean ± standard error (SEM). The 50% inhibitory concentration (IC_50_) was estimated by means of a linear regression equation. Statistical significance was determined by one-way ANOVA followed by Tukey´s multiple comparison test, while lifespan and paralysis assays were calculated using Kaplan–Meier survival analyses with log-rank test. Differences with *p* ≤ 0.05 were considered statistically significant.

## 3. Results

### 3.1. Extract Yield

The extract was prepared from fresh flowers of *B. officinalis*. The yield of the extract was 5.45% (mass of extract/mass of fresh flowers).

### 3.2. Fatty Acid Composition

The results of the main fatty acids (FA) found in the studied *B. officinalis* flower extract, as well as their relative percentages of saturated fatty acids (SFA), monounsaturated fatty acids (MUFA), and polyunsaturated fatty acids (PUFA), are shown in Table 1. In total, twenty-four FA were identified in the *B. officinalis* flower extract. PUFA were the most predominante group of FA, with 55% approximately. The major fatty acid found was linoleic acid (53.7%), followed by palmitic acid (17.17%) and oleic acid (15.93%). For the nutritional value estimation, the atherogenicity index (*AI*) was 0.26. The Cox value was calculated by considering the percentages of unsaturated FA present in the extract. Higher unsaturated FA led to an increase in Cox values. The values are presented in Table 1.

### 3.3. In Vitro Antioxidant Activity Assays (DPPH·Assay, FRAP, and ORAC)

Three different methods were used to assess the antioxidant activity of *B. officinalis* flower extract. Results are shown in Table 2.

Selected standard compounds such as gallic acid (1 µg/mL) or ascorbic acid (3 µg/mL) were also measured to detect the scavenger capacity of *B. officinalis*. IC_50_ values were calculated by non-linear regression for DPPH, reaching 646 µg/mL. The reducing activity was established by FRAP assay and represented as µmol Fe ^2+^/g extract; data were extrapolated and 4 µmol Fe^2+^/g extract was achieved for this extract. Finally, the ORAC assay resulted in 0.54 µmol TE/mg extract.

### 3.4. Neuro-2a Cells Subjected to Oxidative Stress

#### 3.4.1. Neuroprotective Effect of *B. Officinalis* (MTT Assay)

**Figure 1:**
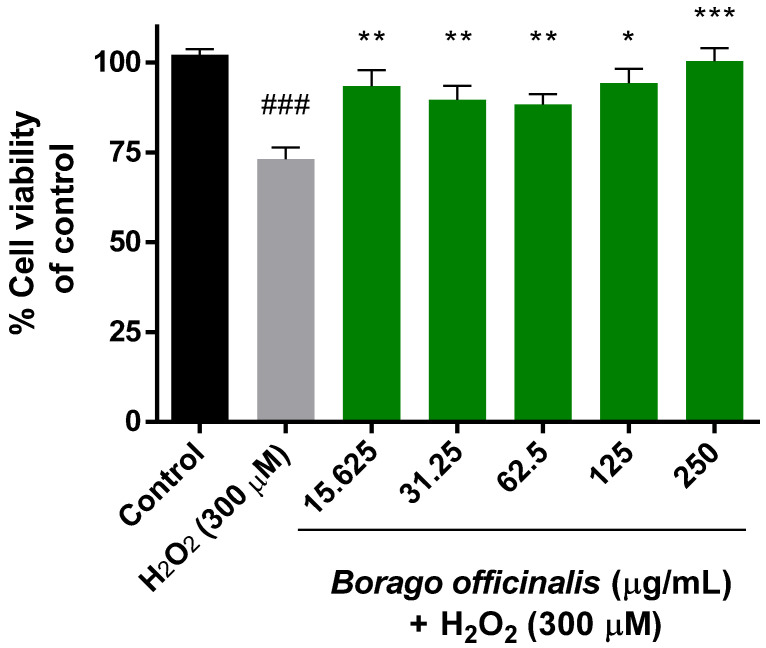
Mitochondrial activity in Neuro-2a cell culture (MTT assay). Cytoprotective effects of *Borago officinalis* flower extract versus hydrogen peroxide (300 µM). Note: * *p* < 0.05 versus H_2_O_2_; ** *p* < 0.01 versus H_2_O_2_; *** *p* < 0.005 versus H_2_O_2_; ### *p* < 0.0005 versus control.

The extensively used MTT assay was required to check the combined effect of hydrogen peroxide and the plant extract over Neuro-2a mitochondria. Previously, the cytotoxicity of the extract was measured for 24 h and none of the concentrations produced any damage to the cells. Once neurons were subjected to hydrogen peroxide (300 µM) for 1 h, the cell viability was reduced by 25% and significant differences were obtained over control cells (Figure 1). In this order, the neuroprotective effect of borago extract was confirmed when cells were subjected to both treatments.

#### 3.4.2. Reactive Oxygen Species (ROS) Production Decreases When Neuro-2a Is Supplemented with *B. officinalis*

**Figure 2:**
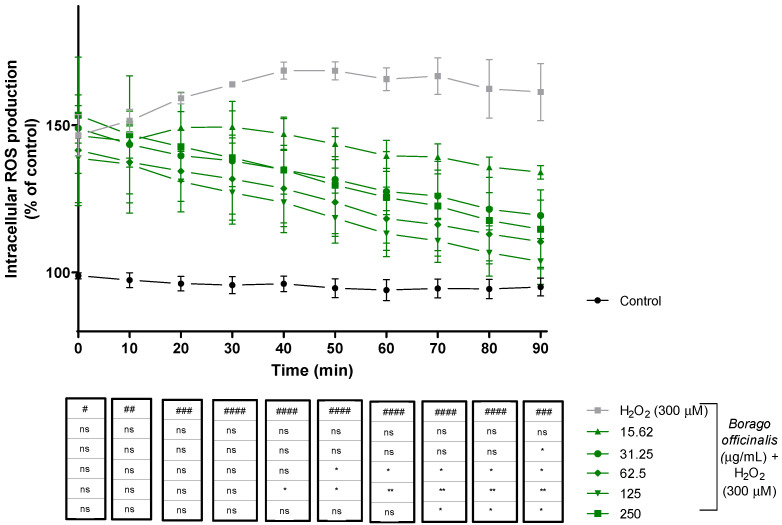
Production of ROS in Neuro-2a cells treated with hydrogen peroxide and *Borago officinalis* flower extract (15.62–250 µg/mL). Data are expressed as percentage over control cells. Note: # *p* < 0.05 versus control; ## *p* < 0.01 versus control; ### *p* < 0.0005 versus control; #### *p* < 0.0001 versus control; ns: no significant; * *p* < 0.05; ** *p* < 0.01. Borage co-treatment did not show any difference until 40 min (125 µg/mL; *p* < 0.05). From this point, ROS species were diminishing for all extract treatments and significant differences were increased. At the end of the experiment, only 15.62 µg/mL did not achieve any significant differences. *Borago officinalis* flower extract (125 µg/mL) reduced ROS production almost to basal levels (control cells).

Intracellular ROS production was measured for 90 min. At the beginning of the assay, treated cells displayed similar ROS production while control cells remained at the same levels during the experiment. *B. officinalis* started to neutralize radicals during the assay, but cells subjected to H_2_O_2_ revealed higher levels (over 150%). In the end, cotreated cells showed values close to the control cells rather than hydrogen peroxide cells, and significant differences appeared (Figure 2).

#### 3.4.3. *B. officinalis* Ameliorates Catalase and Superoxide Dismutase (SOD) Activities

**Figure 3:**
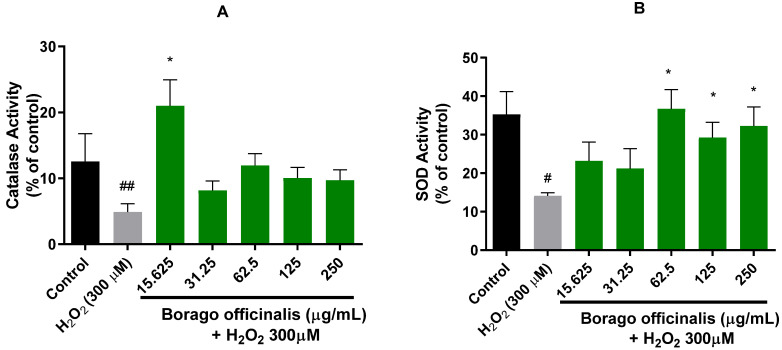
Neuro-2a endogenous antioxidant enzymes exerted by *Borago officinalis* flower extract treatments (15.62-250 µg/mL). (**A**) Catalase activity. (**B**) Superoxide dismutase activity. Note: * *p* < 0.05 versus H_2_O_2_; # *p* < 0.05 versus control; ## *p* < 0.01 versus control.

In order to measure the activity of catalase and superoxide dismutase enzymes, previously, the BCA protein assay was selected, and proteins were normalized. Borage extract demonstrated a similar profile for both enzymes (Figure 3). Remarkably, the lowest concentration expressed the highest activity for catalase, even higher than control cells. The rest of the concentrations retained the activity between control and H_2_O_2_ cells. In the case of SOD activity, the profile showed a slightly dose-dependent tendency. Furthermore, significant differences were detected in the three highest concentrations.

### 3.5. Neurodegenerative Model of C. elegans

#### 3.5.1. Extract of *B. officinalis* Enhances Survival Rate in *C. elegans* Induced with Lethal Oxidative Stress

**Figure 4:**
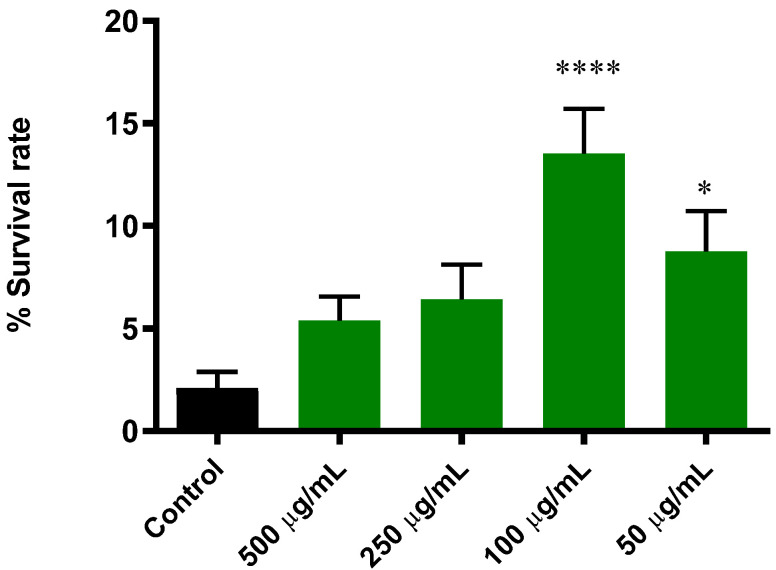
Effect of *B. officinalis* flower extract on the response to lethal oxidative stress induced by juglone (150 μM) on *C. elegans* N2. Differences compared to control group were considered significant at *p* ≤ 0.05 (*) and *p* ≤ 0.0001 (****).

In this study, we used juglone, a powerful redox cycler, to generate lethal oxidative stress [25]. The results in Figure 4 showed a significantly higher survival rate in worms pretreated with *B. officinalis* extracts compared to the control group. The best response to oxidative stress was found in 100 µg/mL, for which the survival rate was increased from 2.1% ± 0.8 (control group) to 13.5% ± 2.1 (*p* ≤ 0.0001).

#### 3.5.2. *B. officinalis* Flower Extract Extends the Lifespan of *C. elegans*

**Figure 5:**
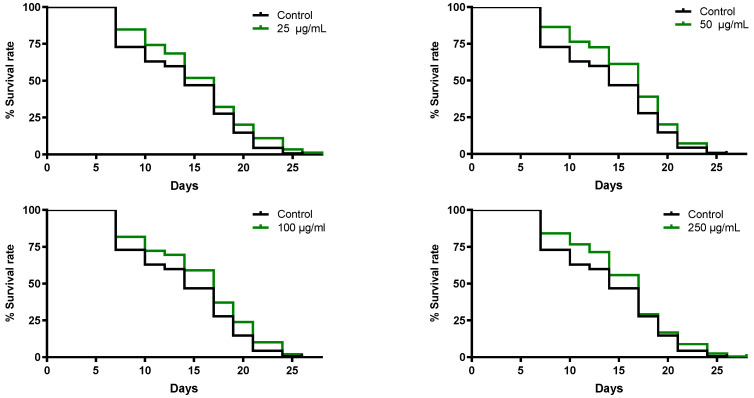
Lifespan of *C. elegans* SS104 subjected to *Borago officinalis* flower extract. The mean lifespan was 14 days in control group and 17 days for all treatment groups. Result were analyzed using the Kaplan–Meier survival model and for significance by using a log-rank pairwise comparison test between the control and treatment groups.

The effect of *B. officinalis* extract on lifespan was assessed in SS104 nematodes, a sterile strain, at a restrictive temperature. By analysis of the survival curves, a significant difference in lifespan extension was observed among the treatment groups and control group (Figure 5). All concentrations tested exhibited a lifespan extension of approximately 18% regarding the control group.

#### 3.5.3. Neuroprotective Effect of *B. officinalis* Flower Extract in *C. elegans*

**Figure 6:**
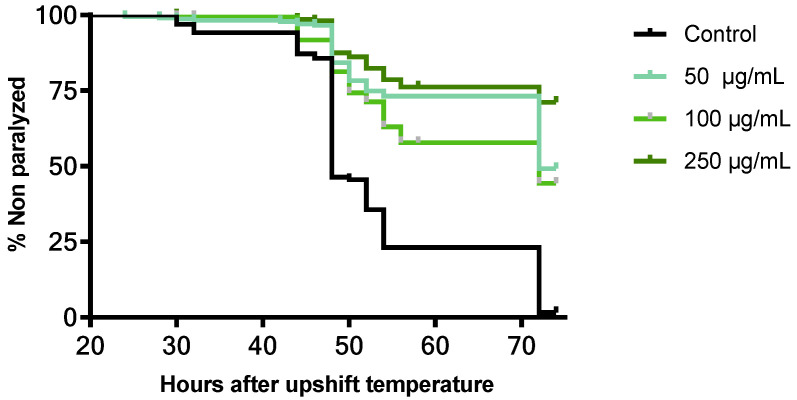
Effect of *B. officinalis* flower extract on Aβ-induced paralysis on transgenic *C. elegans* CL4176. The PT50 were 48 h for the control group and 72 h for 50 and 100 µg/mL treated group. The PT50 was not achieved in the group treated with 250 µg/mL. Statistical significance of the difference between the experiments was analyzed by a log-rank (Kaplan–Meier) statistical test, which compared the survival distributions between the control and treated groups. Differences in survival tests between treatment and the control group were *p* ≤ 0.0001.

The neuroprotective effect of *B. officinalis* flower extract against beta amyloid toxicity was assessed using *C. elegans* CL4176. Figure 6 shows the paralysis curves of CL4176 treated with different concentrations of extract compared with untreated nematodes (control group). After 28 h, after the temperature upshift, the control group started to become paralyzed due to the expression of human β-amyloid peptide. All treatment groups showed a significant delay in body paralysis compared with the control (*p* ≤ 0.0001). The time within which 50% of the worms were paralyzed or dead (PT_50_) was 48 h for the control group, while in treated worms with 50 and 100 µg/mL, it was increased until 72 h after upshifting the temperature. For the highest dose tested, the PT_50_ was not achieved during the experiment.

## 4. Discussion

*B. officinalis* has been previously studied in terms of memory function in animal models using the leaves [26]. In this study, the neuroprotective effect of *B. officinalis* flower extract was assessed on Neuro-2a cells as well as an Alzheimer’s model on *C. elegans*, since there has been an increase in the consumption of edible flowers worldwide.

To perform an appropriate assessment of the antioxidant activity of borage, several methods based on different mechanisms were used: DPPH, FRAP, and ORAC. Previously, free radical scavenging activity was evaluated by using 2,2-diphenyl-1-picryl-hydrazyl-hydrate (DPPH) in other manuscripts. Jaradat et al. described that *Borago officinalis* methanolic extract showed an IC_50_ of 39.92 ± 0.52 μg/mL, while Abu-Qaoud and collaborators studied two different borage cultivars, the wild leaf extract and cultivated leaf extract reaching lower IC_50_ values of 6.3 μg/mL and 8.7 μg/mL, respectively [27,28]. Another study from Italian researchers revealed that an hydroalcoholic extract (70% aqueous EtOH through maceration) from borage leaves achieved 58 μg/mL (IC_50_) [10]. Crude borage methanolic extract displayed 89% inhibition of DPPH radicals by HPLC [9]. All of them demonstrated a potential antioxidant profile for borage, whose values were 20–100 times lower than the one obtained in this manuscript, which suggests that the fresh flowers may have a better antioxidant profile than other parts of the plant due to their composition.

*Borago officinalis*’s ferric reducing antioxidant power (FRAP) was previously determined by an Iranian research group. They also prepared an ethanolic extract and almost reached 3 μmol/g tissue, very similar to the fresh flowers observed in this study (4 μmol/g) [26].

This is the first time that the antioxidant activity of borage has been measured by oxygen radical techniques such as the ORAC assay. This result supports the antioxidant potential evidenced by the previous assays shown in Table 2. Wang et al. carried out a comparison between 12 edible flowers common in China, and reported an ORAC value among 0.86–0.04 µmol TE/mg extract [29]. The ORAC value found in this study for *B. officinalis* flowers is in the upper part of this range.

Despite the scarce literature about the antioxidant activity of the flowers of *B. officinalis*, there are compounds present in it with noted antioxidant activity. As described by Fernandes et al., the flowers of *B. officinalis* are a good source of polyunsaturated fatty acids (PUFAs) and carotenoids; moreover, it contains vitamin E, mainly α-tocopherol [30], and their antioxidant properties are well established [31,32,33].

However, the antioxidant and protective activity in models of oxidative stress in relation to the nervous system has never been established before for these flowers. It is commonly known that neurodegenerative diseases are induced at least in part by free radicals; for this reason, the neuroprotective effect was evaluated in two different models: Neuro-2a and *C. elegans*. No previous paper has studied the effect of this extract in any in vitro neuronal or glial cell line, making it attractive for testing. Hydrogen peroxide was added to the neurons as a free radical initiator and *B. officinalis* neutralized its effect and confirmed the antioxidant activity of the extract. Consequently, the role that it plays as a neuroprotective agent was studied by evaluating the response of the physiological antioxidant defense systems, such as catalase or superoxide dismutase and intracellular ROS production, against hydrogen peroxide.

Borage supplementation reversed the synaptic plasticity in the hippocampal dentate gyrus following Aβ treatment, and thus borage consumption may lead to an improvement in AD-induced cognitive dysfunction [34].

Furthermore, the potential of *B. officinalis* flower extract was challenged on *C. elegans*, which has proven to be a useful in vivo system to study the bioactivity of nutraceuticals and functional foods due to its short life, ease of handling, wide range of mutant strains, and, more importantly, the high degree of similarity with human genetics (between 60 and 80% of human genes have homologues in the worm´s genome) [35]. Initially, the antioxidant potential of *B. officinalis* against induced lethal oxidative stress was tested using juglone, a strong prooxidant, to stress the worms. High doses of juglone generate superoxide and H_2_O_2_, which causes protein modifications, lipid oxidation, and nuclear DNA damage in *C. elegans* [36]. The studied extract can alleviate juglone’s toxicity since it increased the survival rate. Moreover, *B. officinalis* promoted lifespan extension in *C. elegans*. To the best of our knowledge, this is the first study of *B. officinalis* on *C. elegans* regarding its antioxidant and anti-ageing capacity. Some of the compounds described in the flowers of *B. officinalis* are already reported in the literature for protecting worms against oxidative stress and extending their lifespan, such as polyunsaturated fatty acids (PUFAs) [30,32]. Our results agree with these findings and support the use of this species as a functional ingredient with a lack of toxicity.

Experimental and clinical evidence suggests that oxidative stress plays a crucial role in the pathogenesis of Alzheimer’s disease and other neurogenerative disorders [37,38]. In CL4176, prior to the appearance of symptoms of Alzheimer’s disease (paralysis in nematodes), there is an accumulation of oxidative damage that could be attenuated due to the antioxidant activity showed by the extract [39]. The results obtained from paralysis assays showed a significant delay in the time to develop paralysis in a dose-dependent manner. On the other hand, this finding could also be explained by the capacity of different extracts of *B. officinalis* shown to inhibit amyloid formation in vitro and in vivo [34].

These results can be explained partially by the high amount of polyunsaturated FAs (PUFA) found in the extract. Several authors consider that FAs are essential for neurogenesis and neurotransmitter production [40,41,42,43]. We can also highlight that the IA in FAs in borage extract was 0.26. According to Montaner et al. [44], this index can range from 0.18 to 0.43, and it is important to consider the different effects that FAs might have on human health, particularly the probability of decreasing the incidence of pathogenic effects of atheroma and brain inflammatory processes. Regarding the calculated oxidizability value (Cox) of 5.73, the tendency of borago flower extract to prevent oxidative deterioration was observed, which supports its significant dietary nutraceutical value.

## 5. Conclusions

Our study showed that the fresh flowers of *B. officinalis* exhibit neuroprotective and antioxidant activity in vitro as well as in vivo. The studied extract was able to decrease ROS production and increase antioxidant enzyme activity in neuronal cells. Moreover, *B. officinalis* attenuated Aβ toxicity in an Alzheimer’s model of *C. elegans*. Interesting results are here presented; nevertheless, more in vitro experiments in human cells and in vivo models are needed to confirm and develop *B. officinalis* flowers as functional ingredients for human use.

## Figures and Tables

**Table 1:** Fatty acid composition of *B. officinalis* flower extract.

Fatty Acids	Shorthand Nomenclature	Relative Percentage (%)
Caproic acid	6:0	0.24 ± 0.04
Caprylic acid	8:0	0.0802 ± 0.0001
Capric acid	10:0	0.041 ± 0.003
Docosanoic acid	22:0	1.39 ± 0.001
Dodecanoic acid	12:0	0.114 ± 0.002
Heptadecanoic acid	17:0	0.48 ± 0.01
Lignoceric acid	24:0	0.732 ± 0.001
Myristic acid	14:0	0.45 ± 0.04
Pentadecanoic acid	15:0	2.48 ± 0.03
Palmitic acid	16:0	17.17 ± 0.04
Stearic acid	18:0	3.8 ± 0.1
Tricosylic acid	23:0	0.32 ± 0.01
Eicosenoic acid	20:1	0.49 ± 0.02
Erucic acid	22:1 ω 9	0.16 ± 0.01
Myristoleic acid	14:1	0.24 ± 0.01
Nervonic acid	24:1	1.05 ± 0.01
Oleic acid	18:1 ω 9	15.93 ± 0.04
Palmitoleic acid	16:1	0.19 ± 0.01
Eicosadienoic acid	20:2	0.42 ± 0.04
Eicosatrienoic acid	20:3 ω 3 + 21:0	0.276 ± 0.004
Eicosapentaenoic acid	20:5 ω 3	0.12 ± 0.01
γ-Linolenic acid	18:3 ω 6	0.13 ± 0.01
α-Linolenic acid	18:3 ω 3	0.07 ± 0.01
Linoleic acid	18:2 ω 6	53.7 ± 0.2
Σ Saturated fatty acids (SFA)		27.27
Σ Monounsaturated fatty acids (MUFA)		18.05
Σ Polyunsaturated fatty acids (PUFA)		54.68
Σ ω 6		53.83
Σ ω 3		0.59
Cox value		5.73
Atherogenicity Index (*AI*)		0.26

**Table 2:** Antioxidant activity of *B. officinalis* flower extract. The results are presented as mean ± SEM.

	DPPH IC_50_ (µg/mL)	FRAP µmol Fe^2+/^g Extract	ORAC µmol TE/mg Extract
*B. officinalis*	646 ± 35	4 ± 2	0.54 ± 0.03

Abbreviation: TE = Trolox equivalent.

## Data Availability

Data is contained within the article.
